# Supplementary material for: Restraining Quiescence Release-Related Ageing in Plant Cells: A Case Study in Carrot
Source: Cells. 2023 Oct 16;12(20):2465. doi: 10.3390/cells12202465 (PMC10605352; doi:10.3390/cells12202465)
Supplement: Supplementary file 1 [file cells-12-02465-s001.zip › Supplementary Figure S1.pptx]

## Slide 1
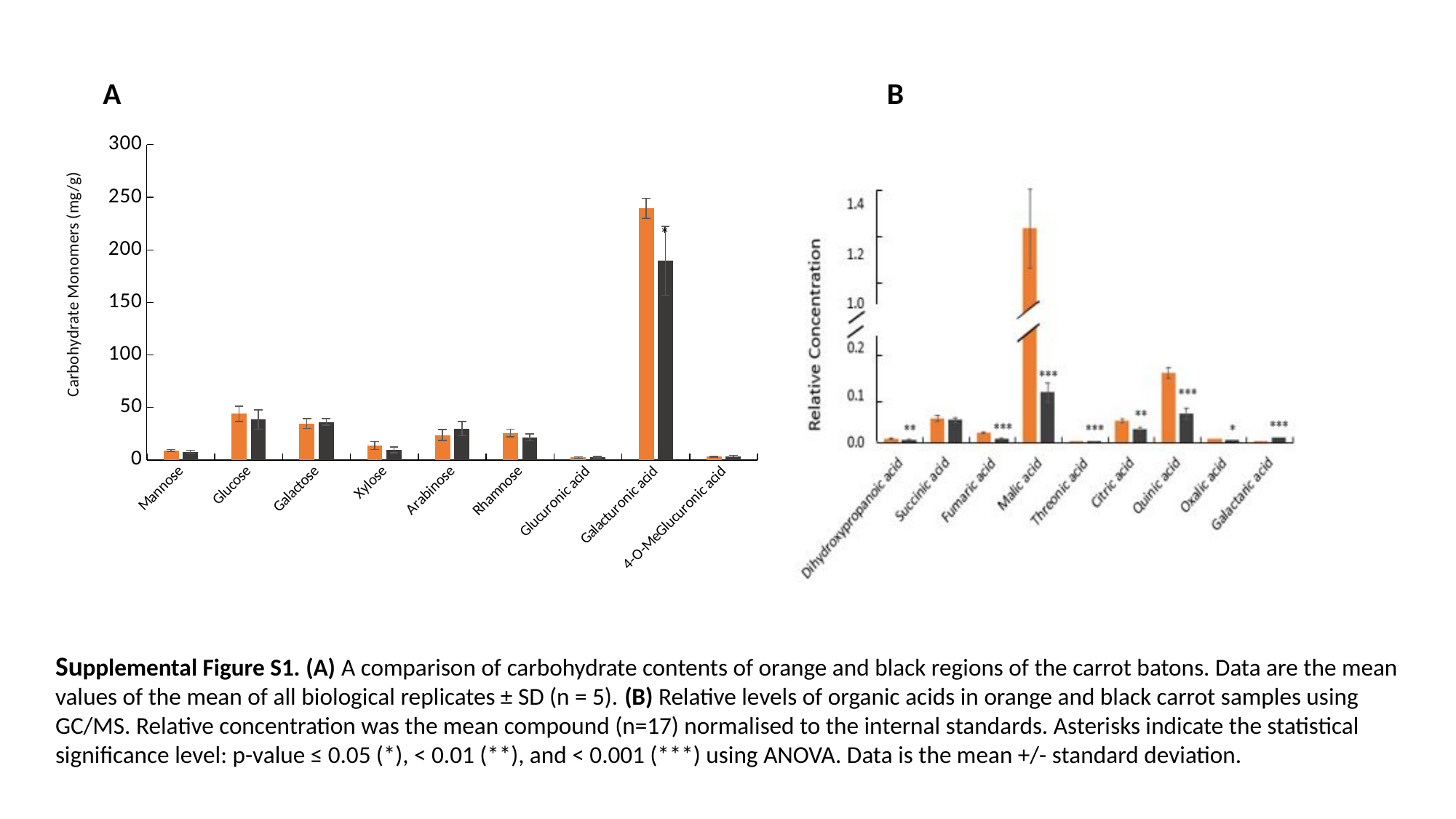

A B
### Chart
| Category | Orange carrot | Blackened carrot |
|---|---|---|
| Mannose | 9.183807899580426 | 7.888349319394441 |
| Glucose | 43.98814468394863 | 38.60052771597968 |
| Galactose | 34.74403227053067 | 36.249569605650976 |
| Xylose | 13.79385117887538 | 9.666424878948671 |
| Arabinose | 23.786225041475713 | 29.90541845354369 |
| Rhamnose | 25.64284457983148 | 21.58634320650914 |
| Glucuronic acid | 2.4955237645730435 | 2.7606710712153566 |
| Galacturonic acid | 239.41547295498032 | 189.48524761035392 |
| 4-O-MeGlucuronic acid | 3.216318855683208 | 3.7394068808234593 |
Supplemental Figure S1. (A) A comparison of carbohydrate contents of orange and black regions of the carrot batons. Data are the mean values of the mean of all biological replicates ± SD (n = 5). (B) Relative levels of organic acids in orange and black carrot samples using GC/MS. Relative concentration was the mean compound (n=17) normalised to the internal standards. Asterisks indicate the statistical significance level: p-value ≤ 0.05 (*), < 0.01 (**), and < 0.001 (***) using ANOVA. Data is the mean +/- standard deviation.
