# Supplementary material for: Restraining Quiescence Release-Related Ageing in Plant Cells: A Case Study in Carrot
Source: Cells. 2023 Oct 16;12(20):2465. doi: 10.3390/cells12202465 (PMC10605352; doi:10.3390/cells12202465)
Supplement: Supplementary file 1 [file cells-12-02465-s001.zip › Supplementary Figure S10.pptx]

## Slide 1
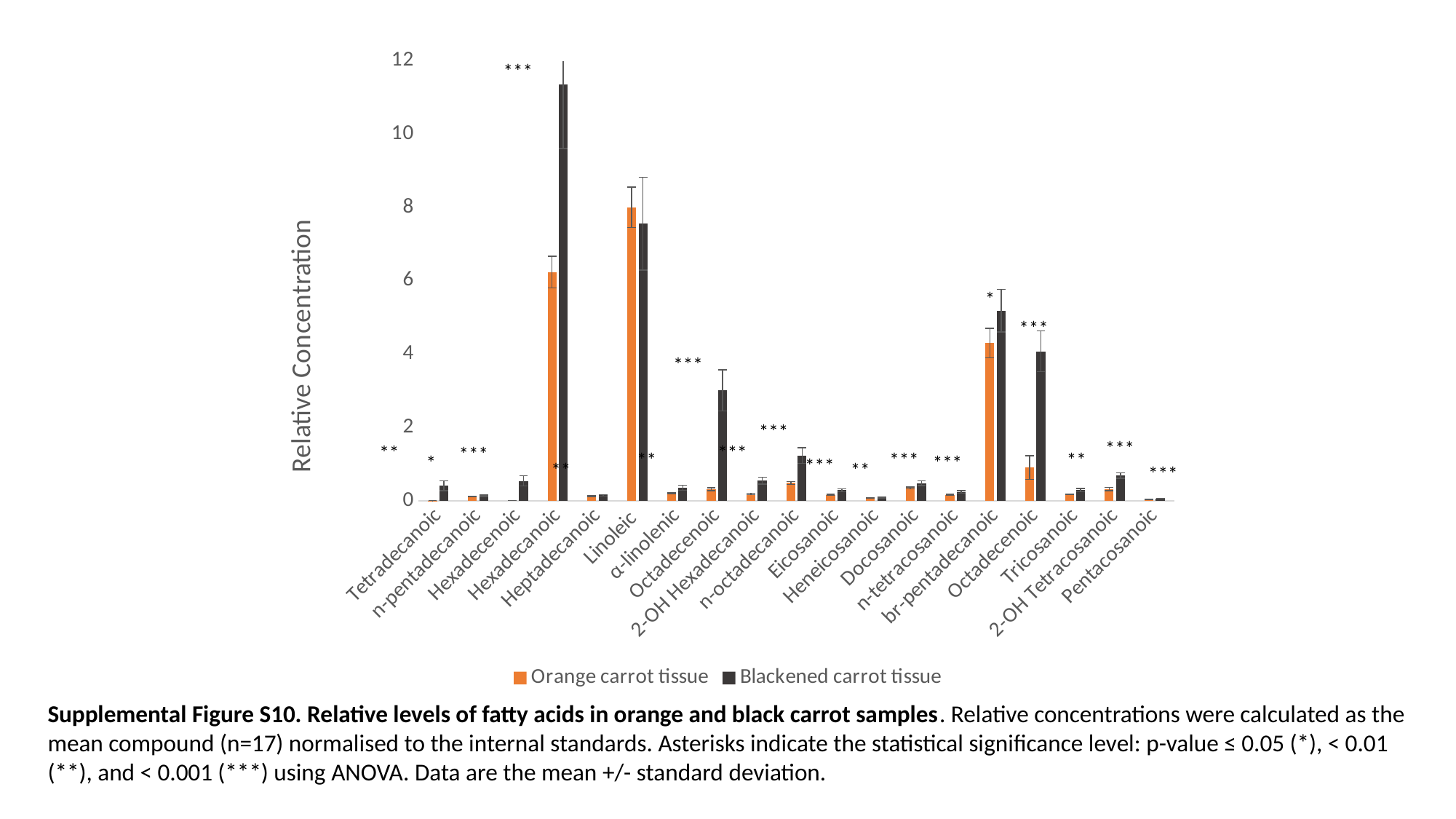

### Chart
| Category | Orange carrot tissue | Blackened carrot tissue |
|---|---|---|
| Tetradecanoic | 0.004403462079997698 | 0.40670091487815413 |
| n-pentadecanoic | 0.11681937602552207 | 0.1390109503574878 |
| Hexadecenoic | 0.008804559114529199 | 0.540856733617903 |
| Hexadecanoic | 6.234687666378624 | 11.367090982682928 |
| Heptadecanoic | 0.12240911915270933 | 0.1476081534248674 |
| Linoleic | 8.004532007920638 | 7.560018300499561 |
| α-linolenic | 0.20984689128812448 | 0.3574949484114895 |
| Octadecenoic | 0.316293218711057 | 3.008316389971631 |
| 2-OH Hexadecanoic | 0.18528512524799712 | 0.5458373732447133 |
| n-octadecanoic | 0.4853207785664768 | 1.2301757469958297 |
| Eicosanoic | 0.16410657106026666 | 0.2853219299443712 |
| Heneicosanoic | 0.0782003182800669 | 0.09345613142980182 |
| Docosanoic | 0.34980712074621784 | 0.48118161758846095 |
| n-tetracosanoic | 0.1577070701387304 | 0.23611183322633925 |
| br-pentadecanoic | 4.3057199247894165 | 5.187824005687486 |
| Octadecenoic | 0.904423535369253 | 4.0777552158676365 |
| Tricosanoic | 0.17291724519779175 | 0.29324935992972784 |
| 2-OH Tetracosanoic | 0.31855832894331915 | 0.6878823108545491 |
| Pentacosanoic | 0.0303155416370847 | 0.05781422912370515 |***
*
***
***
***
***
***
**
***
**
***
**
*
***
***
**
**
***
Supplemental Figure S10. Relative levels of fatty acids in orange and black carrot samples. Relative concentrations were calculated as the mean compound (n=17) normalised to the internal standards. Asterisks indicate the statistical significance level: p-value ≤ 0.05 (*), < 0.01 (**), and < 0.001 (***) using ANOVA. Data are the mean +/- standard deviation.
