# Supplementary material for: Restraining Quiescence Release-Related Ageing in Plant Cells: A Case Study in Carrot
Source: Cells. 2023 Oct 16;12(20):2465. doi: 10.3390/cells12202465 (PMC10605352; doi:10.3390/cells12202465)
Supplement: Supplementary file 1 [file cells-12-02465-s001.zip › Supplementary Figure S2.pptx]

## Slide 1
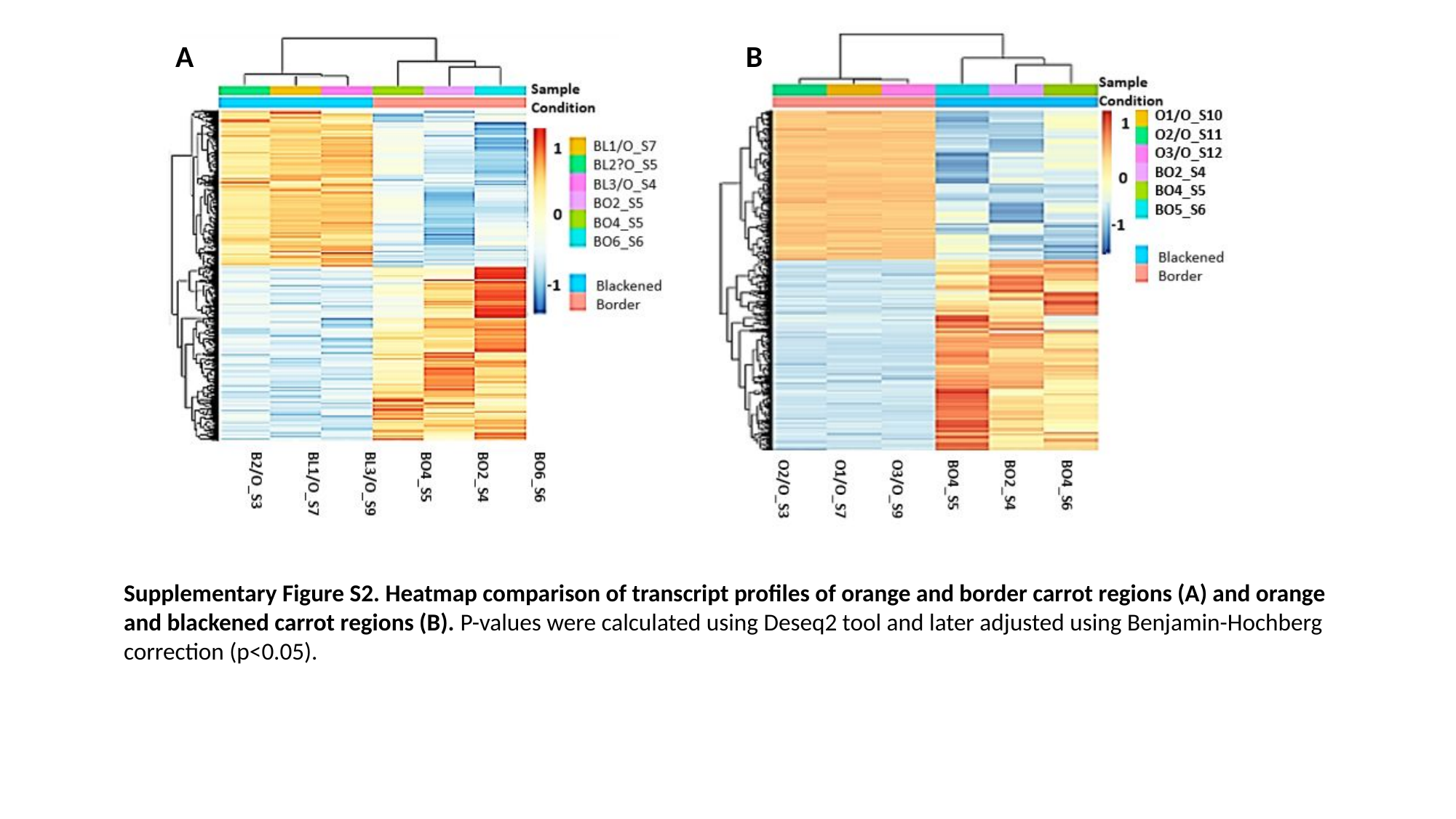

A B
Supplementary Figure S2. Heatmap comparison of transcript profiles of orange and border carrot regions (A) and orange and blackened carrot regions (B). P-values were calculated using Deseq2 tool and later adjusted using Benjamin-Hochberg correction (p<0.05).
