# Supplementary material for: Restraining Quiescence Release-Related Ageing in Plant Cells: A Case Study in Carrot
Source: Cells. 2023 Oct 16;12(20):2465. doi: 10.3390/cells12202465 (PMC10605352; doi:10.3390/cells12202465)
Supplement: Supplementary file 1 [file cells-12-02465-s001.zip › Supplementary Figure S3.pptx]

## Slide 1
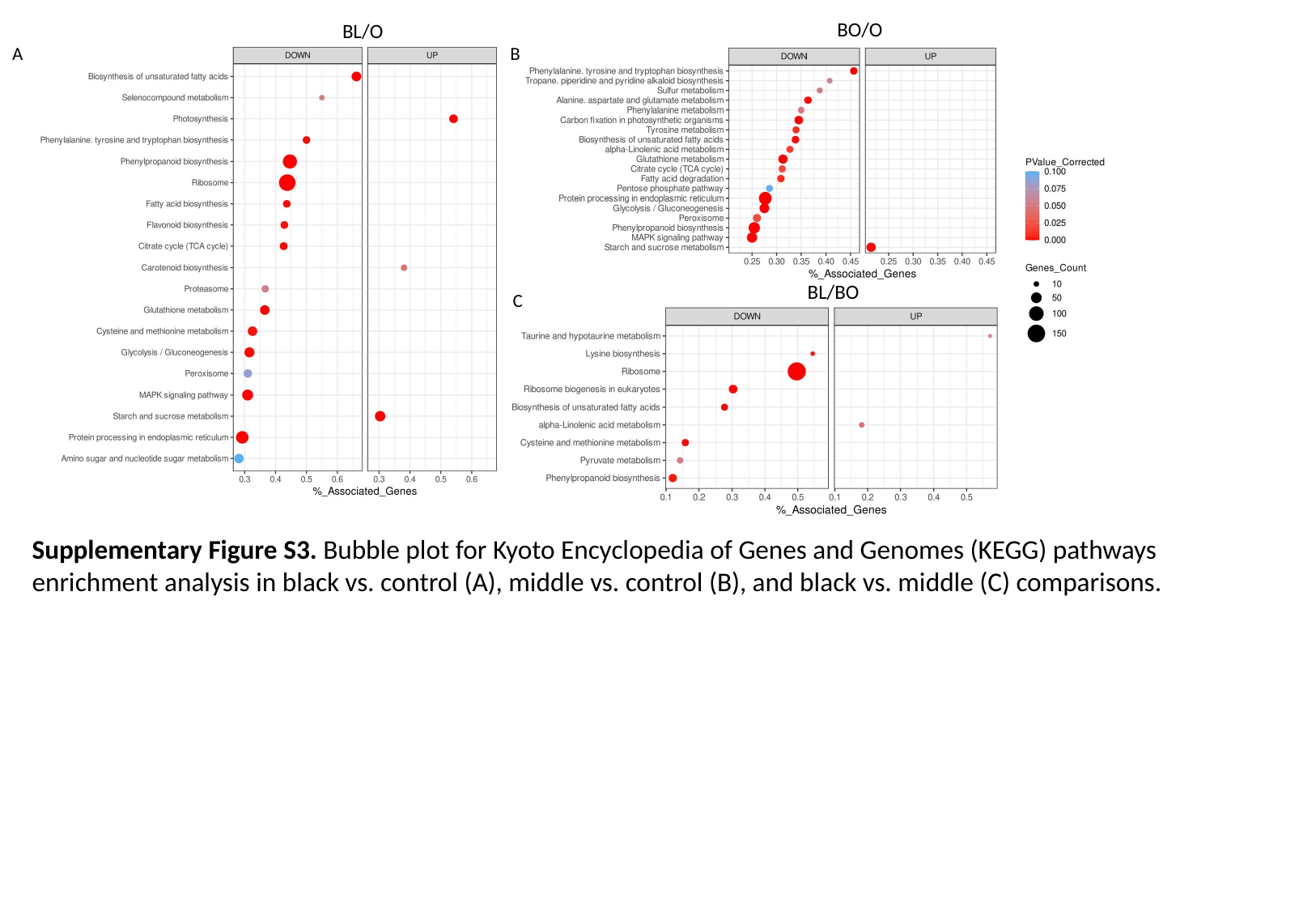

BO/O
BL/O
A
B
BL/BO
C
Supplementary Figure S3. Bubble plot for Kyoto Encyclopedia of Genes and Genomes (KEGG) pathways enrichment analysis in black vs. control (A), middle vs. control (B), and black vs. middle (C) comparisons.
