# Supplementary material for: Restraining Quiescence Release-Related Ageing in Plant Cells: A Case Study in Carrot
Source: Cells. 2023 Oct 16;12(20):2465. doi: 10.3390/cells12202465 (PMC10605352; doi:10.3390/cells12202465)
Supplement: Supplementary file 1 [file cells-12-02465-s001.zip › Supplementary Figure S4.pptx]

## Slide 1
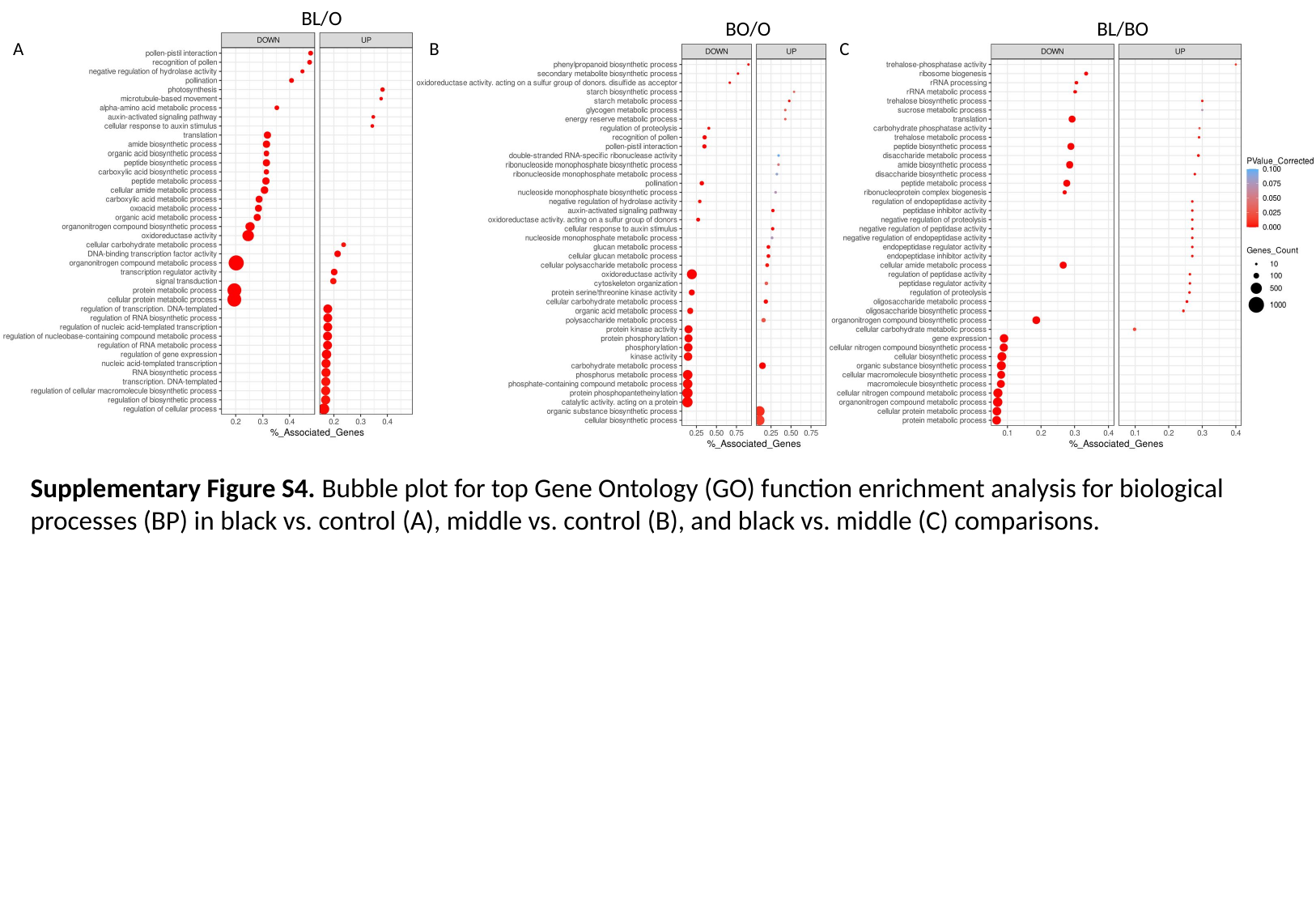

BL/O
BL/BO
BO/O
A
B
C
Supplementary Figure S4. Bubble plot for top Gene Ontology (GO) function enrichment analysis for biological processes (BP) in black vs. control (A), middle vs. control (B), and black vs. middle (C) comparisons.
