# Supplementary material for: Restraining Quiescence Release-Related Ageing in Plant Cells: A Case Study in Carrot
Source: Cells. 2023 Oct 16;12(20):2465. doi: 10.3390/cells12202465 (PMC10605352; doi:10.3390/cells12202465)
Supplement: Supplementary file 1 [file cells-12-02465-s001.zip › Supplementary Figure S5.pptx]

## Slide 1
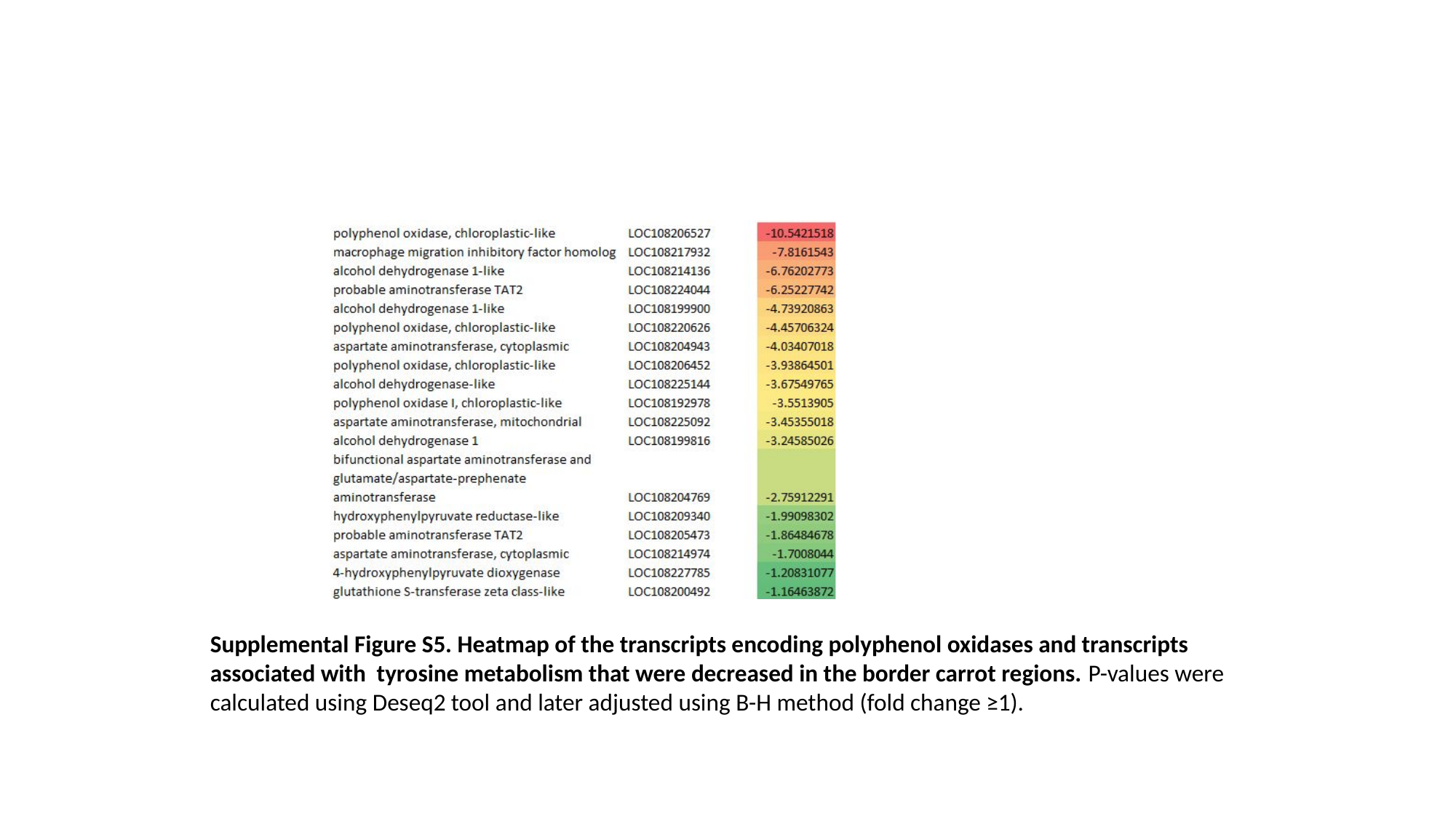

Supplemental Figure S5. Heatmap of the transcripts encoding polyphenol oxidases and transcripts associated with tyrosine metabolism that were decreased in the border carrot regions. P-values were calculated using Deseq2 tool and later adjusted using B-H method (fold change ≥1).
