# Supplementary material for: Restraining Quiescence Release-Related Ageing in Plant Cells: A Case Study in Carrot
Source: Cells. 2023 Oct 16;12(20):2465. doi: 10.3390/cells12202465 (PMC10605352; doi:10.3390/cells12202465)
Supplement: Supplementary file 1 [file cells-12-02465-s001.zip › Supplementary Figure S6.pptx]

## Slide 1
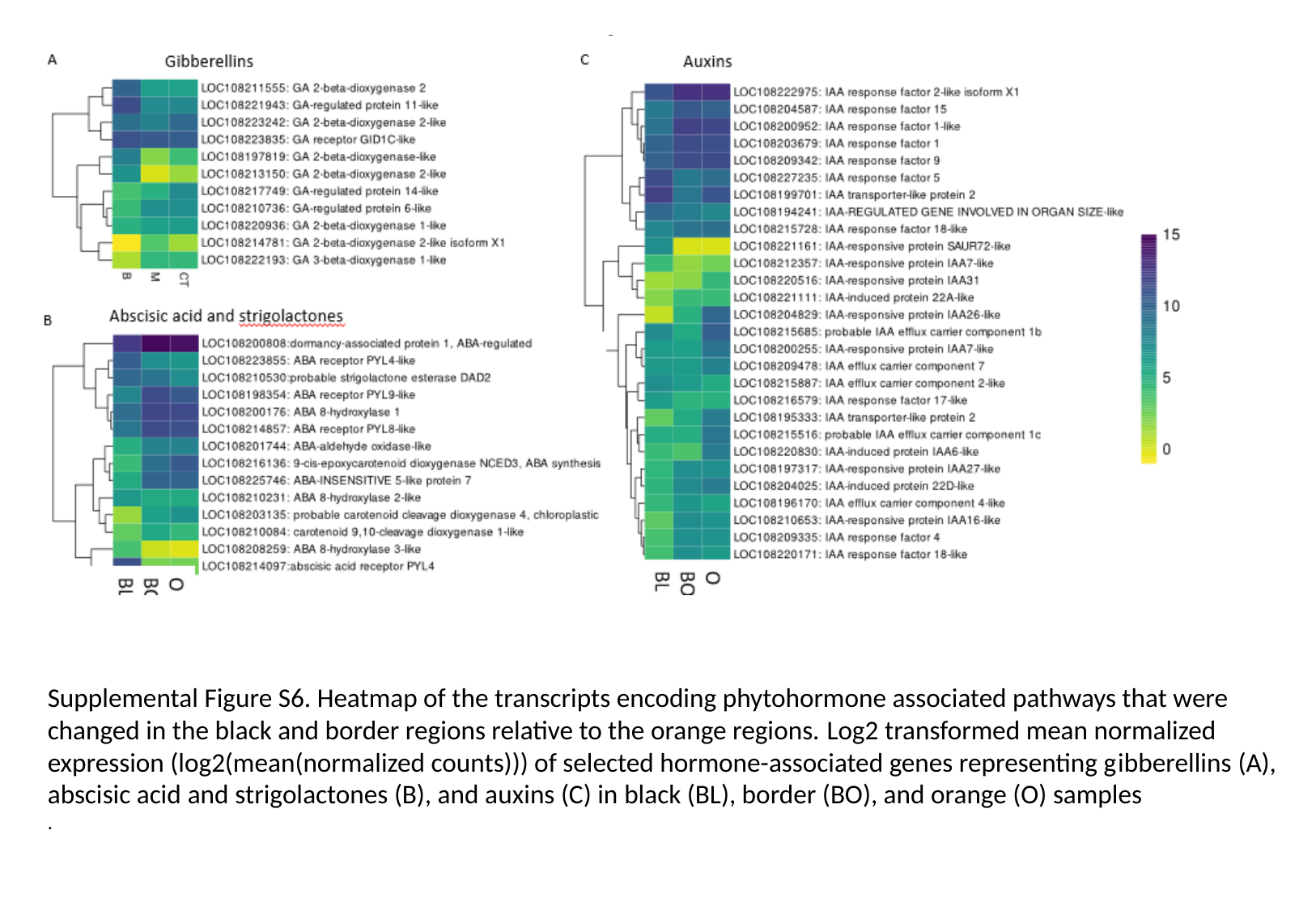

C
Supplemental Figure S6. Heatmap of the transcripts encoding phytohormone associated pathways that were changed in the black and border regions relative to the orange regions. Log2 transformed mean normalized expression (log2(mean(normalized counts))) of selected hormone-associated genes representing gibberellins (A), abscisic acid and strigolactones (B), and auxins (C) in black (BL), border (BO), and orange (O) samples
.
