# Supplementary material for: Restraining Quiescence Release-Related Ageing in Plant Cells: A Case Study in Carrot
Source: Cells. 2023 Oct 16;12(20):2465. doi: 10.3390/cells12202465 (PMC10605352; doi:10.3390/cells12202465)
Supplement: Supplementary file 1 [file cells-12-02465-s001.zip › Supplementary Figure S7.pptx]

## Slide 1
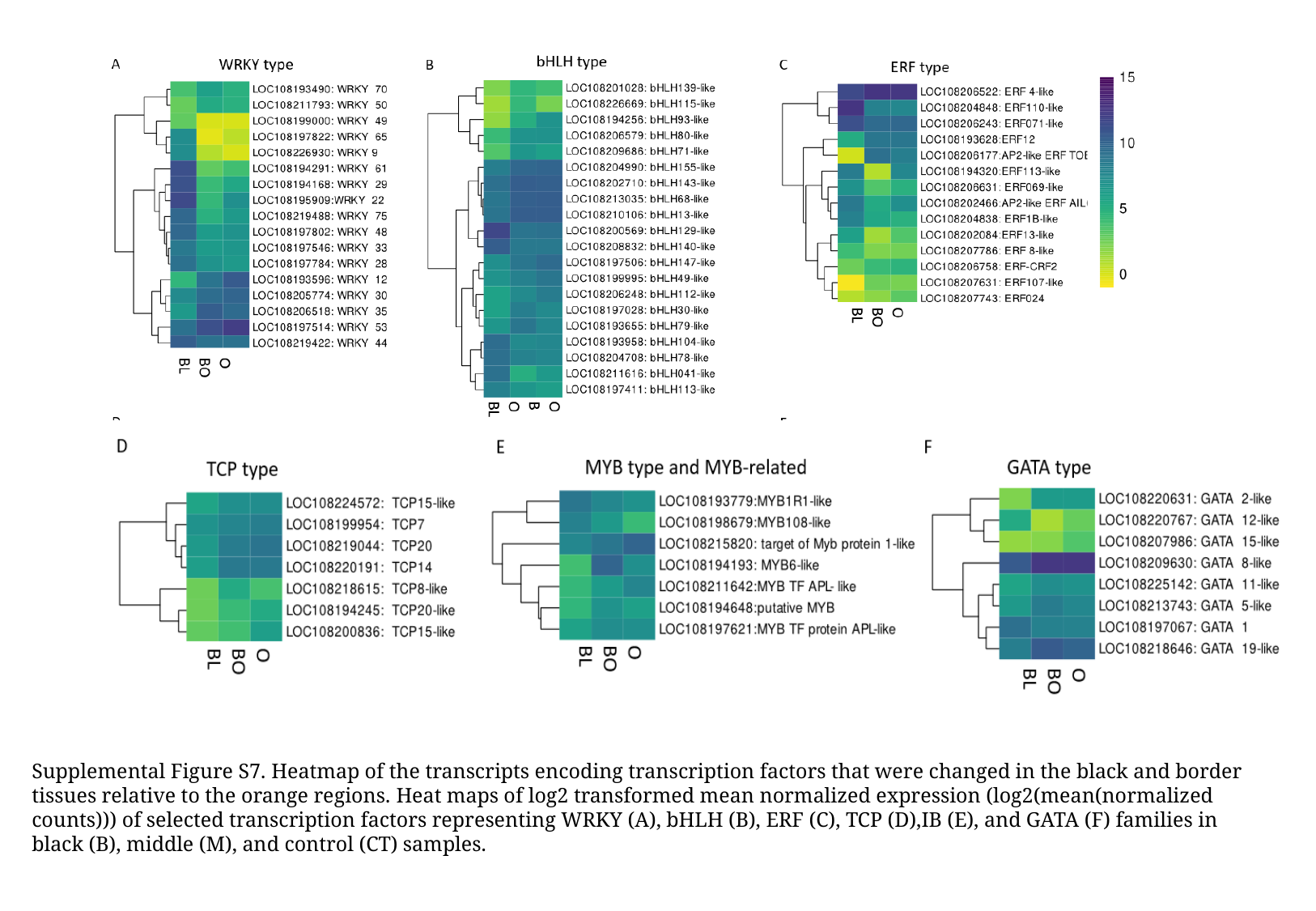

E
F
Supplemental Figure S7. Heatmap of the transcripts encoding transcription factors that were changed in the black and border tissues relative to the orange regions. Heat maps of log2 transformed mean normalized expression (log2(mean(normalized counts))) of selected transcription factors representing WRKY (A), bHLH (B), ERF (C), TCP (D),IB (E), and GATA (F) families in black (B), middle (M), and control (CT) samples.
