# Supplementary material for: Restraining Quiescence Release-Related Ageing in Plant Cells: A Case Study in Carrot
Source: Cells. 2023 Oct 16;12(20):2465. doi: 10.3390/cells12202465 (PMC10605352; doi:10.3390/cells12202465)
Supplement: Supplementary file 1 [file cells-12-02465-s001.zip › Supplementary Figure S8.pptx]

## Slide 1
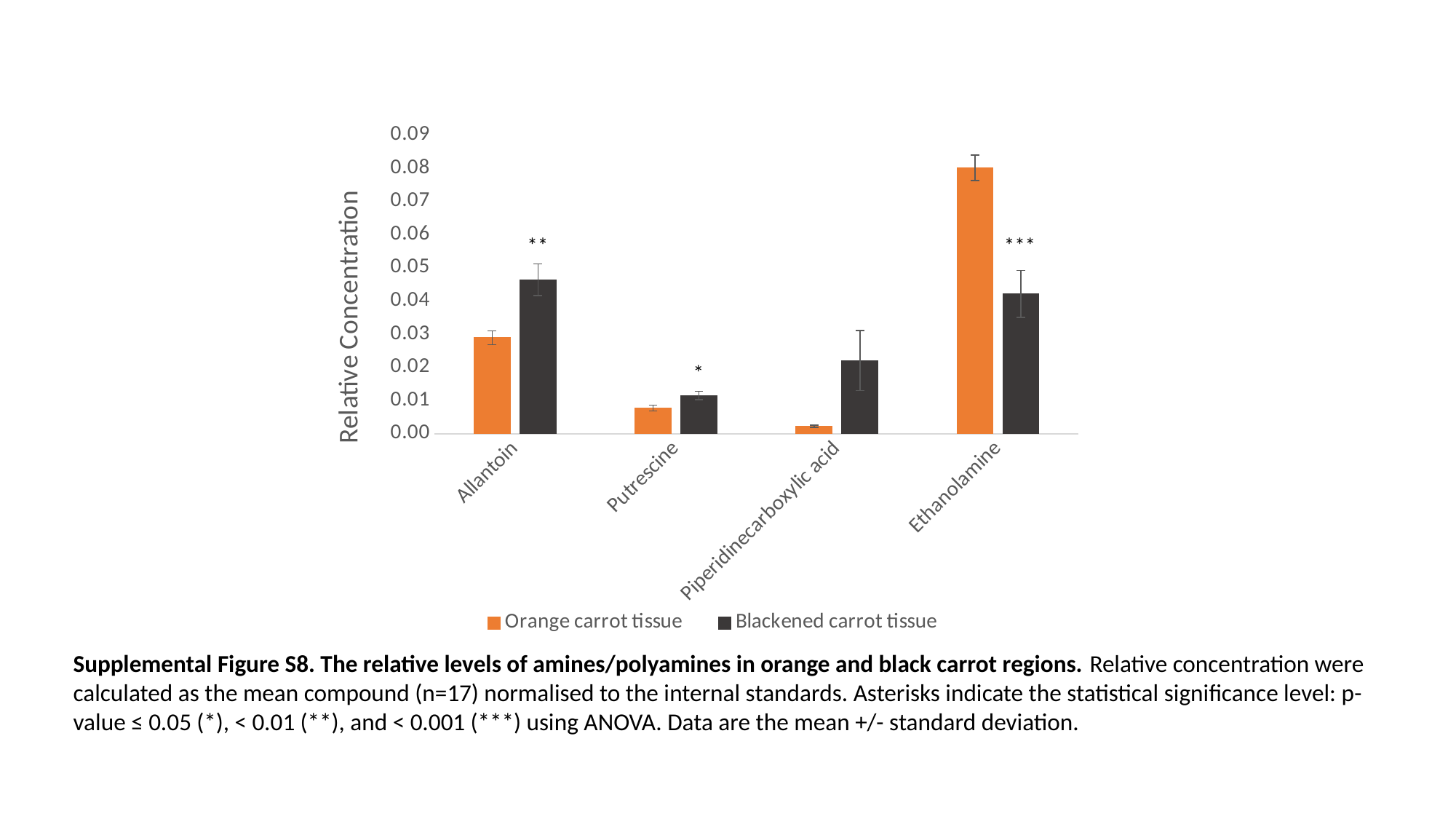

### Chart
| Category | Orange carrot tissue | Blackened carrot tissue |
|---|---|---|
| Allantoin | 0.028927550793824468 | 0.0464202146156083 |
| Putrescine | 0.007756105738678354 | 0.011521965404632822 |
| Piperidinecarboxylic acid | 0.0023043497015062943 | 0.022077263339563644 |
| Ethanolamine | 0.08011390503181241 | 0.04215115274168965 |**
***
*
Supplemental Figure S8. The relative levels of amines/polyamines in orange and black carrot regions. Relative concentration were calculated as the mean compound (n=17) normalised to the internal standards. Asterisks indicate the statistical significance level: p-value ≤ 0.05 (*), < 0.01 (**), and < 0.001 (***) using ANOVA. Data are the mean +/- standard deviation.
