# Supplementary material for: Restraining Quiescence Release-Related Ageing in Plant Cells: A Case Study in Carrot
Source: Cells. 2023 Oct 16;12(20):2465. doi: 10.3390/cells12202465 (PMC10605352; doi:10.3390/cells12202465)
Supplement: Supplementary file 1 [file cells-12-02465-s001.zip › Supplementary Figure S9.pptx]

## Slide 1
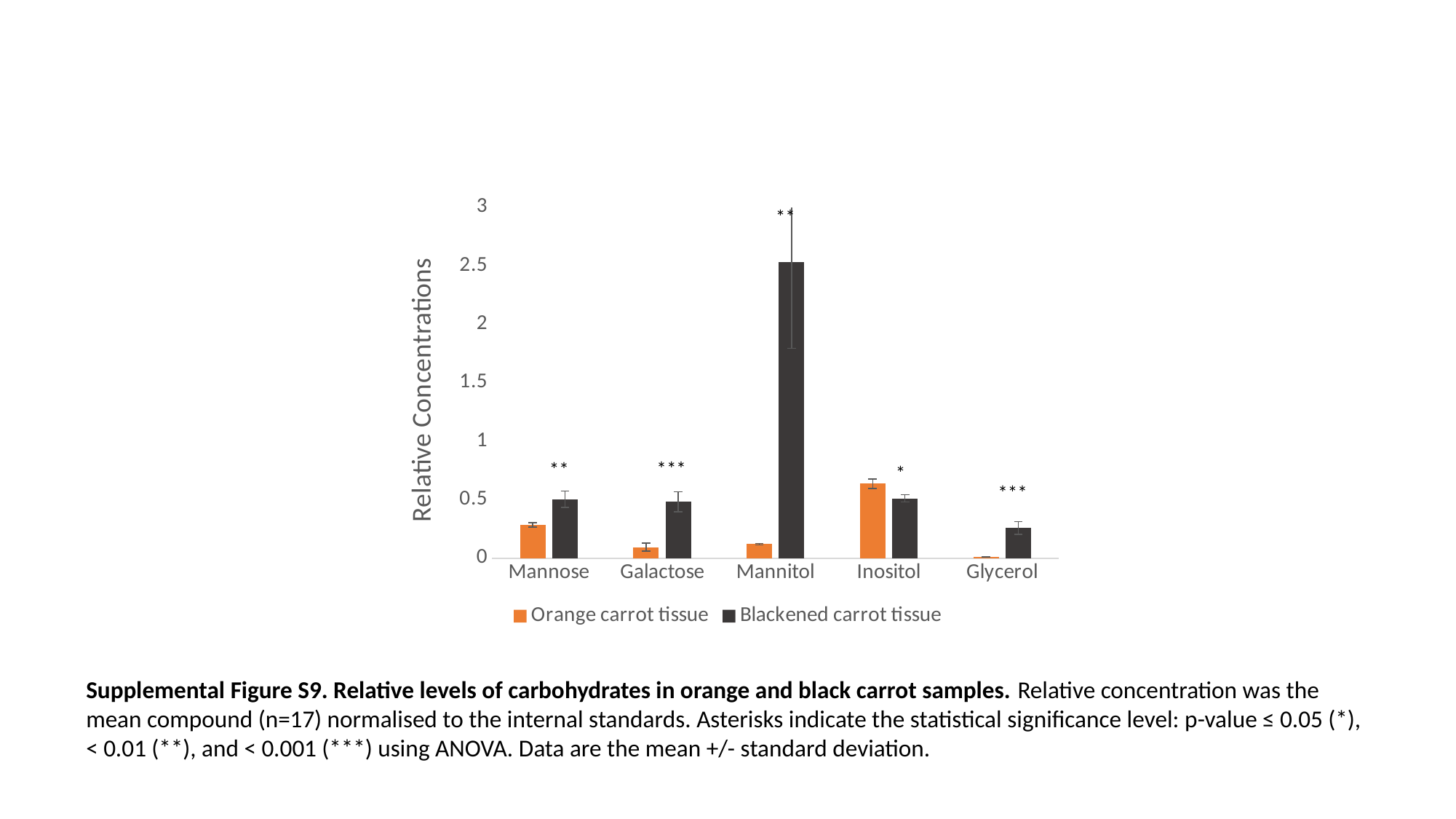

### Chart
| Category | Orange carrot tissue | Blackened carrot tissue |
|---|---|---|
| Mannose | 0.2854531245015951 | 0.5037099513014002 |
| Galactose | 0.09419131160274005 | 0.4828501330605559 |
| Mannitol | 0.12127684249351722 | 2.5315955272720574 |
| Inositol | 0.6367176699646763 | 0.511586409157387 |
| Glycerol | 0.010297133624840051 | 0.25911968364954996 |**
***
**
*
***
Supplemental Figure S9. Relative levels of carbohydrates in orange and black carrot samples. Relative concentration was the mean compound (n=17) normalised to the internal standards. Asterisks indicate the statistical significance level: p-value ≤ 0.05 (*), < 0.01 (**), and < 0.001 (***) using ANOVA. Data are the mean +/- standard deviation.
